# Behavioral guidance for improving dental care in autistic spectrum disorders

**DOI:** 10.3389/fpsyt.2023.1272638

**Published:** 2023-11-14

**Authors:** Irene Pastore, Elena Bedin, Giulia Marzari, Francesca Bassi, Claudio Gallo, Carla Mucignat-Caretta

**Affiliations:** ^1^Community Dentistry Department, Ospedale Immacolata Concezione, Piove di Sacco, Italy; ^2^Department of Statistical Sciences, University of Padova, Padova, Italy; ^3^Department of Molecular Medicine, University of Padova, Padova, Italy

**Keywords:** autism, dentistry, behavior, visual aid, oral health, child support

## Abstract

**Introduction:**

Autism spectrum disorders (ASDs) impair many aspects of everyday life and may prevent access to dental care, often limiting it to emergencies. Impaired oral health has long-lasting negative consequences on health status and on the acquisition of oral habits (e.g., oral respiration and grinding) or competencies (e.g., proper speech production). Children with ASD may be scared in the dental setting, which is rich in sensory stimuli and requires physical contact. Due to their behavioral manifestations, they represent a challenge for dentists and hygienists. We created a dedicated pathway with behavioral support for children with ASD to allow dental care and possibly limit the use of general anesthesia.

**Methods:**

We evaluated the effects of behavioral support in a quasi-experimental design by comparing two groups of children with ASD. The first group (*n* = 84) was visited every 2 months for 3 years and received additional support (visual aids, caregiver training, and longer visit duration). A control group, matched for age and sex, was visited at least twice a year or more, if needed, according to standard healthcare guidelines.

**Results:**

Compliance with the schedule was high throughout the 3 years. The degree of collaboration significantly improved after 1 year in the supported group, while the control group did not change. At the end of the study, collaboration remained significantly higher than at the beginning in the supported group. Half of dental treatments were possible without general anesthesia in supported children. No adverse effect was apparent on collaboration due to COVID-19 restrictions.

**Discussion:**

Behavioral techniques improved the compliance of ASD children to regular dentistry visits and treatment. Furthermore, oral hygiene at home was similarly improved, addressing oral health from a lifelong perspective.

## 1. Introduction

The prevalence of autism spectrum disorders (ASDs) is increasing with similar prevalence worldwide, reaching 1 in 100 children (1, 2). The onset of behavioral symptoms precedes the 2nd year of life. ASDs impact development and the entire life by not only affecting communication skills and social relationships, but also movement and cognition (3, 4). ASDs are accompanied by intellectual disability in approximately one-third of cases and most often affect the male sex, with a male/female ratio of approximately 4.2 (1). ASDs may also be associated with epilepsy or genetic diseases (5, 6). Finally, this condition strongly affects the psychological wellbeing of parents and caregivers (7). Children with ASD represent a challenge for healthcare professionals, in particular dentists because of the prolonged physical contact and invasiveness of the procedures during the visit and treatment (8). However, oral health is a prerequisite for properly acquiring and maintaining oral functions such as mastication, deglutition, proper respiration, and speech production. These functions affect a variety of processes, from ingestion of food—and hence overall health—to language acquisition, cognitive development, and social life. Therefore, caregivers and health workers should recognize early signs of distress in oral issues and provide ASD patients with access to healthcare, including specifically trained dentists (9). While a diffused need for improving oral health in children with ASD is emerging worldwide, most literature on dental care in neurodevelopmental disorders is devoted to epidemiology (10). Autism impacts access to dental care and the development of proper oral hygiene because of difficulties in communication, altered sensitivity, and behavioral conundrums (11), even if the prevalence of malocclusion (12) and decayed, missing, and filled teeth index (13) were found to be similar to control subjects. Children with ASD may present not only dental caries but also traumatic injuries (14), sometimes self-inflicted, saliva drooling or bruxism (15), and a higher plaque index (16). Although very scarce data on the co-occurrence of craniofacial anomalies and ASD are present in the literature, children with those anomalies present a higher prevalence of ASD (17); hence, it is worth considering the possible involvement of connective tissue in ASD pathogenesis (18). Dental treatment aims to correct the acquisition and maintenance of oral functions. Furthermore, in children, conservative treatments and tooth extractions should be preferentially done at the dentist's chair, with the aid of local anesthesia and/or mild sedation if needed (19). Highly invasive cures, such as oral surgery, gingivoplasty, complex conservative treatments, endodontics, or extractions, particularly of the posterior teeth, may require general anesthesia, which nowadays appears to be the first choice for special needs children (20).

At variance with many other fields in health sciences, most articles on dentistry for ASD children come from a variety of countries from all over the world, underlining the spread of this issue throughout human cultures. Sadly enough, even recent studies sustain the use of physical restraint for persons with intellectual disabilities or general anesthesia as the other option (21). However, while restraint is unethical in our view, general anesthesia bears a biological cost, in particular when dealing with the developing central nervous system, and should be used with care when repeated treatment is necessary. It is therefore advisable to implement a method for improving lifelong dental care by overcoming fear and oppositional behavior (22).

Since the literature on the efficacy of psychological techniques for the dental management of autistic patients is scarce (23), mostly with a small sample size and without a control group (24), it may be worth implementing different techniques for improving oral health in ASD children.

Moreover, most of the available data come from surveys completed by parents/caregivers and suggest great difficulty for caregivers and dentists in dealing with ASD patients (25). As a result, toothache is the most frequent, while routine care is the least frequent reason to visit the dentist (26). Parents/caregivers also report difficulty in tooth brushing in more than half of cases (27), a high prevalence of caries (28), and a fearful approach to dental workers, increased by COVID-19 restrictions (29). However, parents' rating of children's behavior is often inconsistent with objective observation (30); hence, it is mandatory to ask for parents' collaboration while still retaining a clinical objective assessment.

Behavioral interventions are highly effective in improving a variety of social deficits (31) and represent the most attractive, even if not the simplest, strategy to increase overall health status in ASD patients. This study aimed to estimate the efficacy of a dedicated pathway for ASD children to improve their behavior, gain access to dental care, and comply with the visits in comparison with the usual care provided at our hospital's Dentistry Department. Visual pedagogy, parent training, a strict bimonthly schedule for appointments spanning over 3 years, and a longer duration of dedicated examination were set up for a group of ASD children, whose behavior was compared with that of children seen on a regular 6-monthly schedule. We hypothesized that a dedicated pathway would allow ASD children to overcome the fear of dentist chairs and accept invasive maneuvers that require prolonged physical contact. Accordingly, we designed a bimonthly schedule for visits over 3 years, supplemented by caregiver training, visual pedagogy instruments to be used at both the hospital and home, and a 1-h duration of visits, longer than usual, during which children could interact with the personnel, caregivers, and the environment.

## 2. Methods

### 2.1. Study design and participants

This study was approved by the Ethical Committee of the Province of Padova, CESC Code 4578/U6/18, according to Italian law. It was carried out at the Community Dentistry Department (Supplementary Figure S1) of the “Immacolata Concezione” Public Hospital in Piove di Sacco (Padova), Italy. The project started on 1 January 2019 and ended on 31 December 2021 and involved 170 children with ASD (see Supplementary Figure S2) in a quasi-experimental design to evaluate the efficacy of the dedicated pathway when compared to standard care. Two groups were enrolled, one whose caregivers were willing to comply with the strict experimental schedule, while the other group followed the usual clinical treatment, according to the caregivers' choice (hence lacking random assignment to groups). The first group of ASD children was selected to enter the protocol (*N* = 86; two quit the project after the first visit), according to the following inclusion criteria: They were 4 to 13 years old at the beginning of the treatment, formally diagnosed and recognized as having autistic spectrum disorder (ASD), in some cases in the frame of genetic syndromes, or pervasive developmental disorder—not otherwise specified (PDD-NOS), a diagnosis present in the Diagnostic and Statistical Manual of Mental Disorders—DSM4—later merged to ASD in DSM5. Exclusion criteria were as follows: age outside the selected range, additional diseases, in particular those preventing autonomous oral hygiene (e.g., motor diseases), familiarity with dental treatments, and good acceptance of dental treatments. Selected patients were not yet regularly seen by dentists, but in some cases, they had received some dental treatment for urgencies before the beginning of the study.

A second group of ASD patients (*N* = 84) served as a control. They were matched 1:1 for age, sex, and diagnosis to the abovementioned group. They were selected for matching the characteristics (age and sex) of the first group on a patient-to-patient basis, among the patients meeting the inclusion criteria of the study, but whose caregivers were not available to adhere to the strict 2-month visit schedule. Patients in this control group were asked to visit at least twice every year, or more if needed, but without a fixed 1-h duration of the visit, visual pedagogy instruments, or parent training. Some children from both groups were scheduled for treatments under general anesthesia when needed from a clinical point of view, without difference in their group affiliation.

### 2.2. Intervention

Caregivers, known by spread of word with the help of associations of parents, were contacted by phone and offered to enter a special treatment plan, involving a 1-h appointment every 2 months for 3 years with the same dentist and professionals in a dedicated environment, and additional support (visual aids and parent training). Caregivers decided whether to stay on the usual treatment (visits every 6 months) or comply with the study requirements. In this case, a map with the pathway from the parking place to the operating room was sent via e-mail, including details of the building (corridors, stairs, and elevators) to familiarize children with the environment. The first physical meeting was devoted to detailed explanations about the project and the signature of informed consent; a weekly diary was also given to take notes about oral hygiene habits; and a plastic mirror was given to be used at home. An anamnestic interview was carried out by the dentist in charge of the case on clinical history, information on therapies (including behavioral) and preferred activities, and the use of positive or negative reinforcers for shaping behavior. The child was set free to explore the environment unless he/she was already cooperative and free to sit in the dentist's chair.

From the second visit, the protocol was adapted to the needs of every child. It included visual pedagogy (if needed) to show the steps for oral hygiene to be tried first in the dental office and then at home. The images (Supplementary Figure S3) were also sent by e-mail to be used at home. If oral hygiene (use of toothbrush and toothpaste) was already an acquired habit, this step was omitted; no change in the habits for oral hygiene was requested if it was done correctly, even if slightly differing from the proposed sequence of actions. If the child entered the operations room, the dentist showed the chair and the instruments; if he/she did not enter, the child was allowed to go to the office, where a table with a computer and some chairs were located. From the third appointment onward, an approach to the visit and possibly professional oral hygiene or dentist treatment was attempted. At every visit, a list of information was reported on a file; see Supplementary Figure S4.

### 2.3. Outcomes

The primary outcome was the degree of collaboration assessed by the dentists (IP and EB, not blind to the treatment) at the end of each visit, measured on the Frankl scale (32), from 0 (no collaboration) to 1 (scarce), 2 (good), and 3 (full collaboration). Collaboration is also operationally described as the total number of accepted instruments or maneuvers. Secondary outcomes are the acceptance or rejection of steps to enter the operating room, of instruments used during hygiene, and of instruments used during dentist operations (see Supplementary Figure S4). We identified the steps for entering the operating room (point 3 in Supplementary Figure S4), all instruments used for dentists' actions (point 4 in Supplementary Figure S4), and professional dental hygiene (point 5 in Supplementary Figure S4). Furthermore, the dental treatments were recorded: sealings, temporary fillings, permanent fillings, and extractions (points 6, 7, 8, and 9, respectively, in Supplementary Figure S4). Data, including positive and negative findings, were collected at the end of each appointment on an encrypted file.

### 2.4. Statistical analysis

The sample size was calculated with G^*^Power 3.1.9.6 (33), with alpha error probability: 0.05, power: 0.85, and estimated effect size: 0.3, returning *n* = 75. We were authorized to add 10 more patients for compensating subjects possibly quitting the study. All subjects were enrolled in the early phases of the study, given its long duration. Data were analyzed with SPSS and PRISM 5 (descriptive statistics, chi-square test, Mann–Whitney and Wilcoxon tests, and linear regression) and Latent GOLD for hierarchical clustering (34). Statistical significance was set at *p* < 0.05, and a 95% CI was used.

The Mann–Whitney and Wilcoxon tests were used to test the difference between and within the experimental and control groups, respectively. The chi-square test was used to analyze the association between categorical variables, and Somers' D was used as a specific measure of association for ordinal variables. Linear regression was used to explore changes in behavior over time. In order to take hierarchical data structure into account, we resorted to multilevel latent class (LC) modeling, which allowed us to identify homogeneous groups of visits similar in the attitude of patients and groups of patients similar in their composition of types of visits. Multilevel LC (MLLC) models perform clustering at both levels of the data, taking into account between- and within-group heterogeneity (35, 36).

Latent class (LC) analysis assumes that one or more latent variables exist and that these variables can be measured through their relationship with observed variables, also known as indicators. LC analysis takes into account the categorical (nominal or ordinal) nature of these variables.

LC analysis can be seen as a model-based method for clustering. It is an interesting alternative to k-means clustering as it is very flexible. It was originally designed for categorical variables, but it can also treat continuous ones; it deals very easily with mixed-scale observed variables. Model specifications and assumptions on parameters can be tested with rigorous statistical tests (35). In the following, we specify an LC model for two-level data. Our data are indeed hierarchical: Visits are level 1 units, and patients are level 2 units. Hierarchical clustering (36) was hence used to identify the type of visits based on the association of behavior; this methodology accounts for the possible correlation existing among level 1 units belonging to the same level 2 units.

The study has been described and reported using the TREND guidelines (37, 38).

## 3. Results

### 3.1. Descriptive statistics

Of the 86 patients initially enrolled (Supplementary Figure S2) to follow the experimental program (Supplementary Figures S3, S4) from 1 January 2019 to 31 December 2021, one male participant and one female participant quit the study after the first visit and were not further considered. In total, 61 patients had an ASD diagnosis, 18 had pervasive developmental disorder—not otherwise specified (PDD-NOS, a diagnosis now merged to ASD), and 3 other diagnoses (e.g., known genetic syndromes in addition to autistic behavior). No significant difference was detected in the diagnoses of male and female participants. A total of 1,440 visits were administered to 84 patients (13 girls and 71 boys), the mean number of visits per patient: 17.14, 95% CI 16.57–17.71 (Supplementary Figure S5), of which 228 visits were delivered to the female participants and 1,212 to the male participants. Dental hygiene visits were 24.9%, dentistry visits were 74.3%, and only 0.8% were visits due to emergencies. During dental hygiene, it was possible to use the contra-angle handpiece with polish in 882 visits (Supplementary Figure S6A). A total of 102 teeth were sealed, and 61 patients had only one tooth sealed (Supplementary Figure S6B), suggesting a good degree of oral hygiene. Temporary filling was done on 37 teeth, and composite filling was done on 31 teeth (Supplementary Figure S6C). Five teeth were extracted, including two baby and three permanent teeth. Local anesthesia was used five times (two for fillings and three for tooth extraction), suggesting a good acceptance of manipulations. Of 84 patients, 26 needed interventions under general anesthesia: 8 (9.5%) before entering the project, 17 during the 3-year project, and 1 both before and during the project. During the project, a total of 172 teeth were treated under general anesthesia in 21.4% of patients (*n* = 18). Hence, in these 84 children with ASD, a total of 342 dental treatments were done, 49.7% of which were in the dentist's chair, in order to cure 37.23% of teeth (a single tooth could receive more than one treatment).

### 3.2. Behavioral analysis

Except for the first visit of only one patient, all patients accepted to enter the hospital, even during the COVID-19 pandemic, which required a more stringent protocol for access involving temperature measurement and wearing facial masks for patients, caregivers, and healthcare professionals. Only in two appointments, the patients refused to enter the consulting room, while support from caregivers was requested in 116 cases (Supplementary Figure S7A). After entering the consulting room, the children were asked to sit in the office, a procedure actively refused in 148 cases (Supplementary Figure S7B), while entering the operating room was refused only 41 times (Supplementary Figure S7C). In 48 visits, the children refused to open the mouth (Supplementary Figure S7D). Once the children entered the operating room, 1,145 visits were done at the chair, while a minority were done standing (*n* = 93), in the caregivers' arm (*n* = 20), or in the pushchair (*n* = 3) (Supplementary Figure S7J). Once the visit started, the different instruments were accepted to a variable degree. The dental mirror and light were refused in 13% of visits (Supplementary Figure S7E), while the towel was refused in 28% of cases (Supplementary Figure S7G). The air-water syringe was refused 32% of the time and the dentist's probe 41% (Supplementary Figures S7I, H, respectively).

### 3.3. Primary outcome: scoping analysis on the degree of collaboration

**Figure 1:**
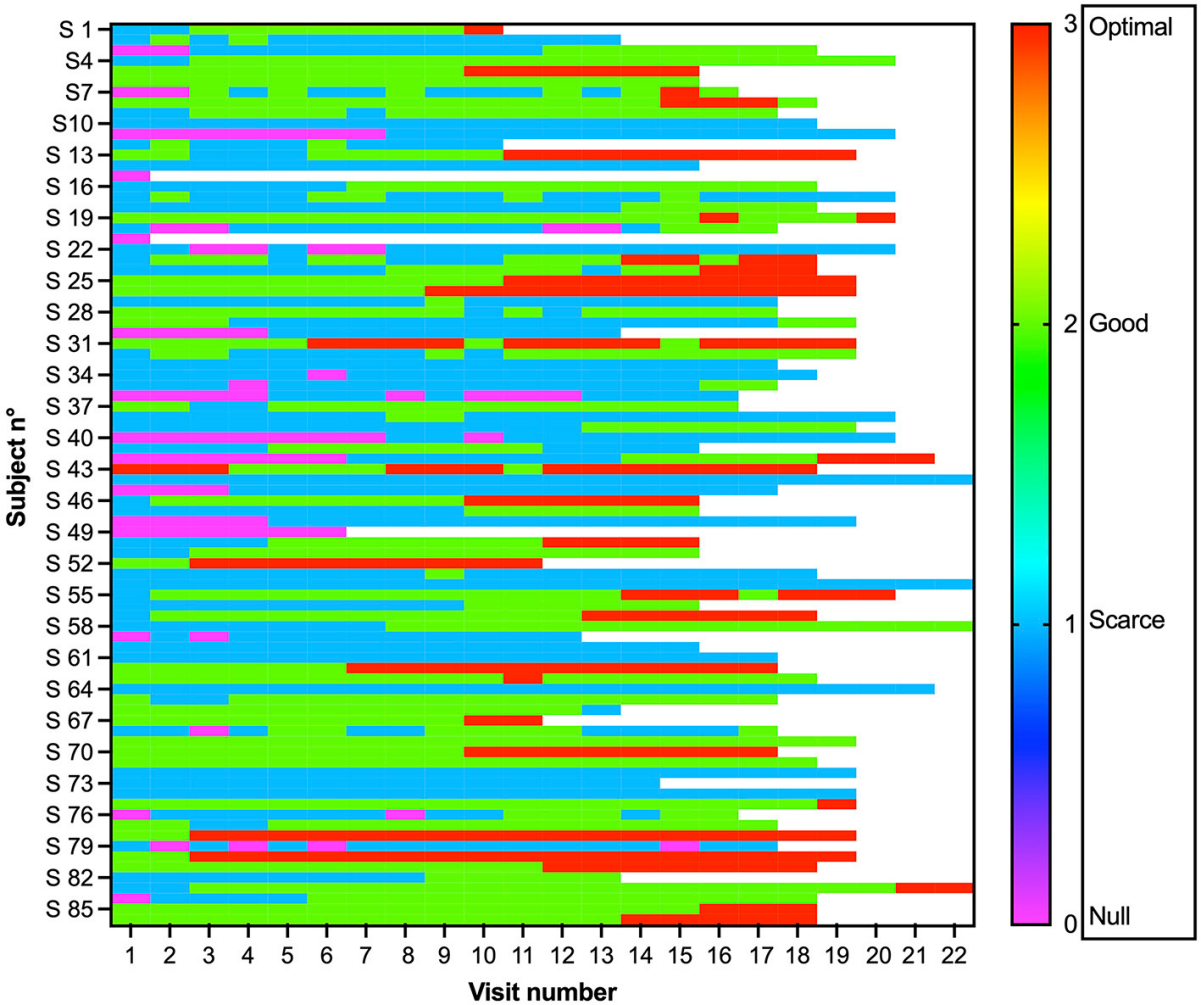
Collaboration shown by each of the 86 patients at each visit. The degree of collaboration was evaluated by the dentist at each visit on a Frankl scale: 0 no collaboration, pink; 1 scarce, light blue; 2 good, green; and 3 optimal, red.

The degree of collaboration at each appointment is shown in Figure 1.

**Figure 2:**
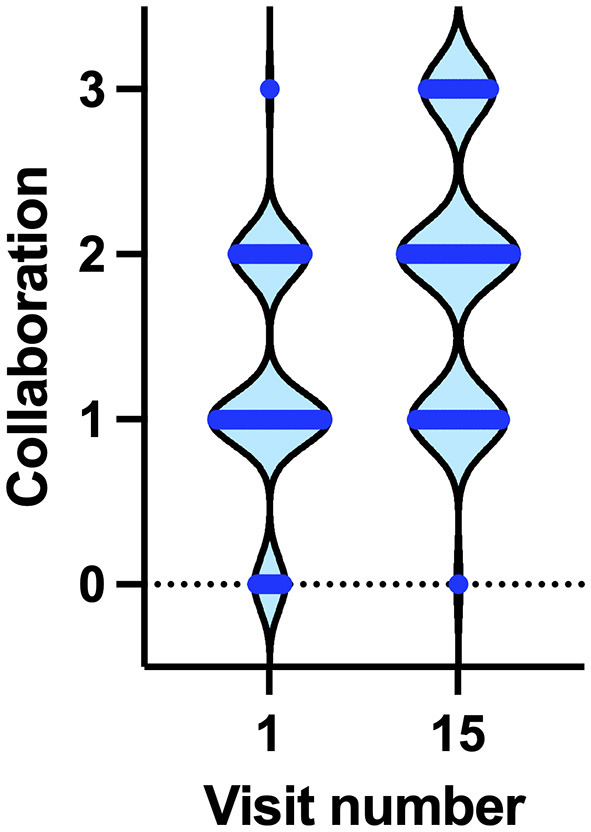
Degree of collaboration at the 1st and 15th visits. Horizontal blue lines indicate the number of patients showing a degree of collaboration from 0 (no collaboration) to 3 (optimal collaboration).

We first asked whether there was a difference in collaboration between the first visit and the last visit. As the total number of visits varied because of possible missing visits or increased frequency for clinical reasons, we chose visit 15 because of possible delays, meaning 5 visits/year and representing an advanced stage in the program that was reached by most of the patients (*N* = 73, 87.95% of patients; see Figure 2, Supplementary Figure S5A). Between the 1st and 15th visits, there was a significant improvement in the degree of collaboration (W = 837, *p* < 0.0001, Wilcoxon test), mean difference (0.671), and CI (0.489–0.854).

Actually, the degree of collaboration varied among the first 15 visits (*p* < 0.0001, Friedman test). Compared with the first visit, the degree of collaboration was significantly higher from visit 9 onward (Dunn's multiple comparisons test). From visit 7 onward, there was no statistically significant difference in collaboration with subsequent visits, suggesting that the improvement in collaboration was reached during the first six visits, which encompasses the first year of treatment. Already at visit 6, the mean value for collaboration was 1.466 (CI 1.300–1.631), while the median improved from 1 at the first visit to 2 at the sixth visit, indicating that 50% of the children reached a good degree of collaboration (*p* < 0.01, Wilcoxon test). However, during the second and third years of the project, the COVID-19 pandemic posed a great threat to autistic persons, possibly delaying or impeding any further possible improvement in collaboration. Nevertheless, it is noteworthy that the achievements of the 1^st^ year were neither lost nor impaired, despite the increased difficulties in accessing the hospital and the department.

### 3.4. Primary outcome: hierarchical clustering analysis on the degree of collaboration

**Figure 3:**
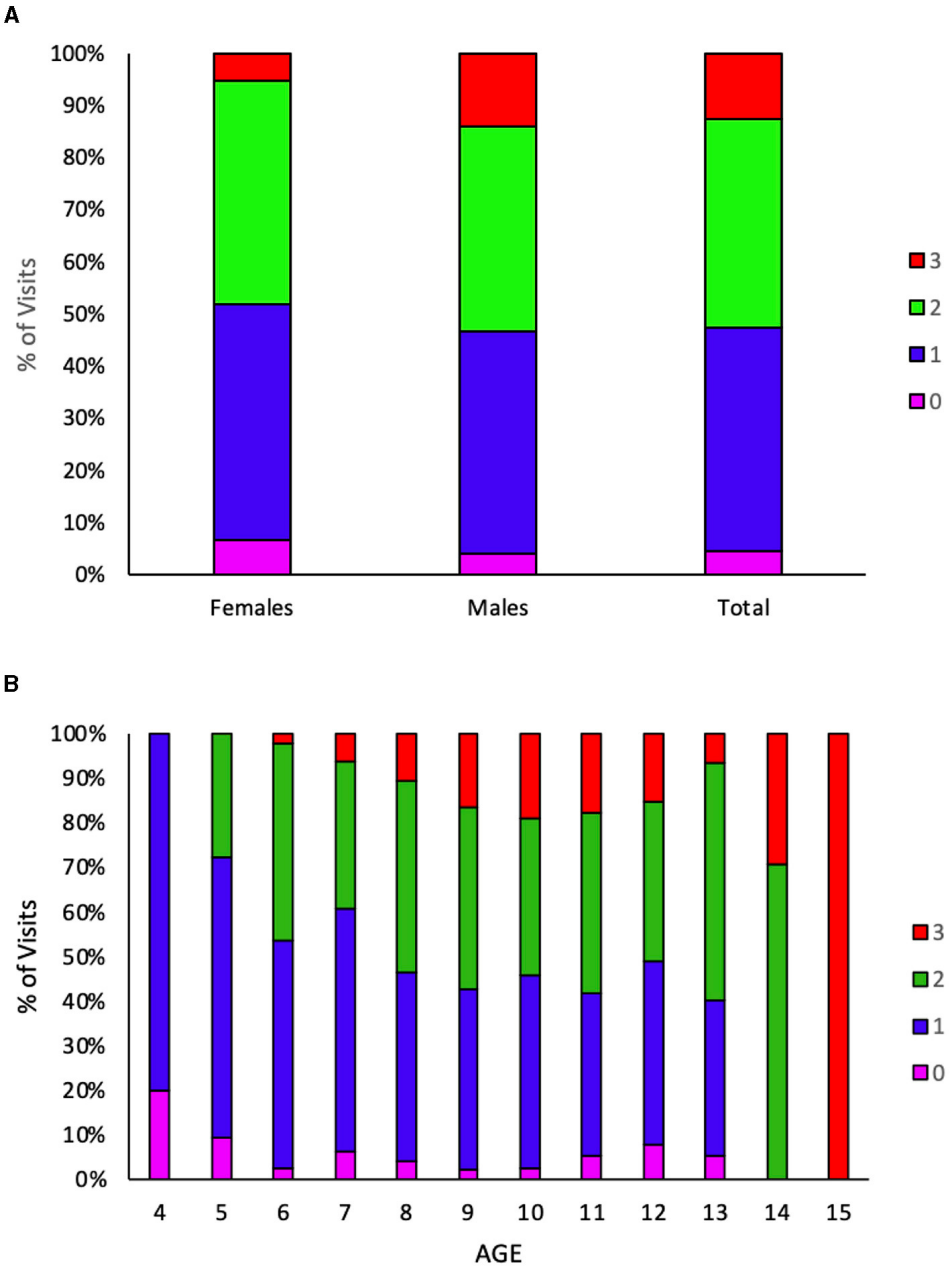
Collaboration varies by sex and age. Collaboration is color-coded. 0 none, pink; 1 scarce, blue; 2 good, green; 3 optimal, red. **(A)** The percentage of visits showing each degree of collaboration is plotted for female participants, male participants, and the whole group. **(B)** The percentage of visits showing each degree of collaboration is plotted at each age, reached by the children at each given visit.

Collaboration was associated with sex (Somers' D: 0.132), being slightly but significantly higher in male participants (chi-square test = 15,469 *p* = 0.01, Figure 3A). Collaboration was also associated with age (Somers' D: 0.298), with the older patients being more collaborative (chi-square test = 181,605 *p* < 0.001, Figure 3B), and diagnosis (chi-square test = 91,004 *p* < 0.001), as children with a diagnosis of ASD are less collaborative. The association between collaboration and age can be in part biased by the fact that during the 3-year project, children were growing older and improving their collaboration as a result of their participation in the project. Concerning diagnosis, the older PDD-NOS (pervasive developmental disorder—not otherwise determined) diagnosis was usually used for symptoms milder than those for autism diagnosis and was often used in toddlers/young children before releasing a clear diagnosis of autism. Hence, it is conceivable that the ASD group includes more severe, and hence less collaborative, cases compared with PDD-NOS.

**Figure 4:**
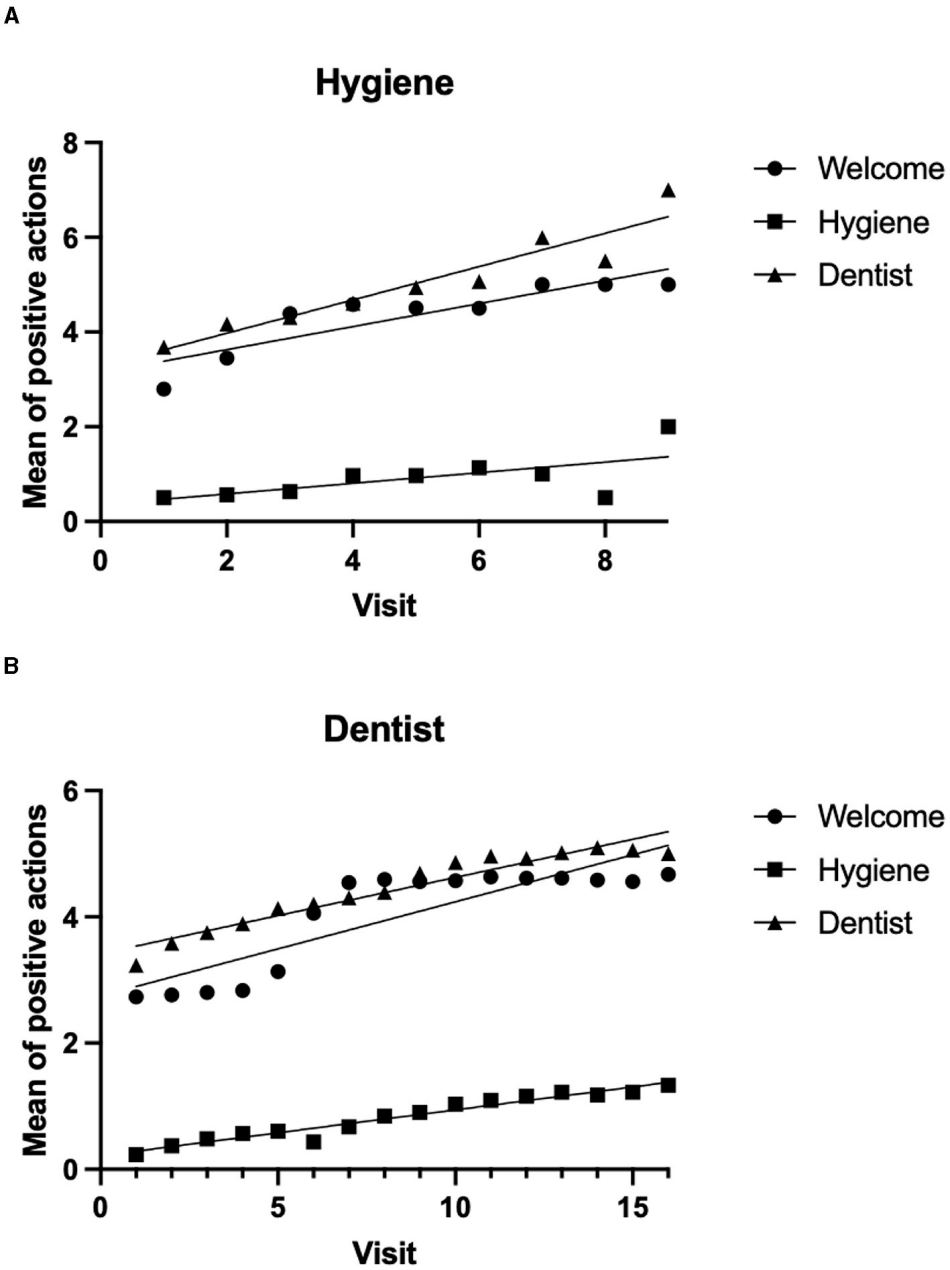
Collaboration, measured as the mean number of accepted actions/instruments, increases with the number of visits. At every visit involving either hygiene **(A)** or dentist visits **(B)**, the number of positive actions or accepted tools was summed for each of the three steps: entrance to the department/rooms and welcome procedures; professional hygiene procedures; dentist visits and procedures. **(A)** Mean of positive actions during appointments for oral hygiene by visit number. **(B)** Mean of positive actions during dentist visits by visit number.

Collaboration was not significantly associated with the type of visit (hygiene or dentistry) (chi-square test = 6.069; *p* = 0.425), indicating that collaboration is not operator-dependent in the present setting. We expected that hygiene could be less invasive than dentist visits. However, when looking at the type of visit, whether hygiene or dental, a similar trend was observed. By summing all the positive responses to all the variables that described the phases of the visits (entering the structure, hygiene procedures, and dentist visits), the mean number of accepted actions increased with the number of visits for both hygiene and dentist visits, as shown by simple linear regression (Figure 4). In all linear regression models, the X variable represents the number of visits. For hygiene visits, the fitted regression model for the welcome procedure was Y = 0.2432^*^X + 3.143, R^2^=0.7743; for hygiene procedures, the model was Y = 0.1122^*^X + 0.3581, R^2^ = 0.4211; and for dentist procedures, Y = 0.3518^*^X + 3.272, R^2^=0.8938. The three slopes (standard error) are as follows: 0.2432 (0.049), 0.1122 (0.358), and 0.3518 (0.045), respectively, and they are significantly different [F_(2, 21)_ = 6.143, *p* < 0.008]. During professional hygiene visits, the increase in the number of accepted actions was significantly different from zero for welcome and dentist procedures [F_(1, 7)_ = 24.02, *p* < 0.002 and F_(1, 7)_ = 58.94, *p* < 0.0001, respectively], but not for hygiene treatments [F_(1, 7)_ = 5.09, *p* = 0.0586]. For dentist visits, the fitted regression model for the welcome procedure was Y = 0.1491^*^X + 2.747, R^2^ = 0.7382; for hygiene procedures, the model was Y = 0.07274^*^X + 0.2143, R^2^ = 0.9476; and for dentist procedures, Y = 0.1209^*^X + 3.416, R^2^ = 0.9237. The three slopes (standard error) are as follows: 0.1491 (0.023), 0.0727 (0.004), and 0.1209 (0.009), respectively; and they are significantly different [F_(2, 42)_ = 6.672, *p* < 0.003]. During dentist visits, the increase in the number of accepted actions was significantly different from zero for welcome procedures, F_(1, 14)_ = 39.49, *p* < 0.0001; for hygiene procedures, F_(1, 14)_ = 252.90, *p* < 0.0001; and for dental treatments, F_(1, 14)_ = 169.5, *p* < 0.0001.

The best-fitting model for hierarchical cluster analysis (Bayesian Information Criterion—BIC index = 15,381) allowed for the identification of four groups of visits, clustered by patients' behavior and groups of similar patients. The classification of visits (Table 1) does not depend on the type of visit, whether hygiene or dentist, but from the order of visits, the collaboration tends to increase in subsequent visits.

**Table 1:** Collaboration-based clustering of visits.

**Cluster**	**4**	**3**	**2**	**1**
% of visits in each cluster	36.06%	25.75%	21.01%	17.18%
Welcome procedures	Very positive or positive collaboration. Accepts all requests. Seats	Positive collaboration. Seats. Accepts all requests except facial mask	Negative collaboration. Enters, seats, but refuses all other requests	Very negative or negative collaboration. Refuses all requests
Hygiene visit	Accepts all requests	Refuses all requests	Refuses all requests	Refuses all requests
Dentist visit	Accepts all requests	Accepts all requests, except toothbrush	Accepts some requests: light, mirror, opens mouth	Refuses all requests

Subsequently, each patient was assigned to one of the four groups according to their behavior during each visit (Table 2). The behavior shown at each visit was not dependent on sex, diagnosis (ASD, PDD-NOS, or other), or the total number of visits; instead, it was linked to age (F = 15,225 *p* = 0.032), with older patients being more collaborative. The effect of age may include developmental issues and the effect of more visits received during the project.

**Table 2:** Clustering of patients according to collaboration degree.

**Type of collaboration**	**4**	**3**	**2**	**1**
% visits	36.06	25.75	21.01	17.18
% patients	89	77	70	83

Each patient was assigned to one of the previously identified types of visits (see Table 1).

**Figure 5:**
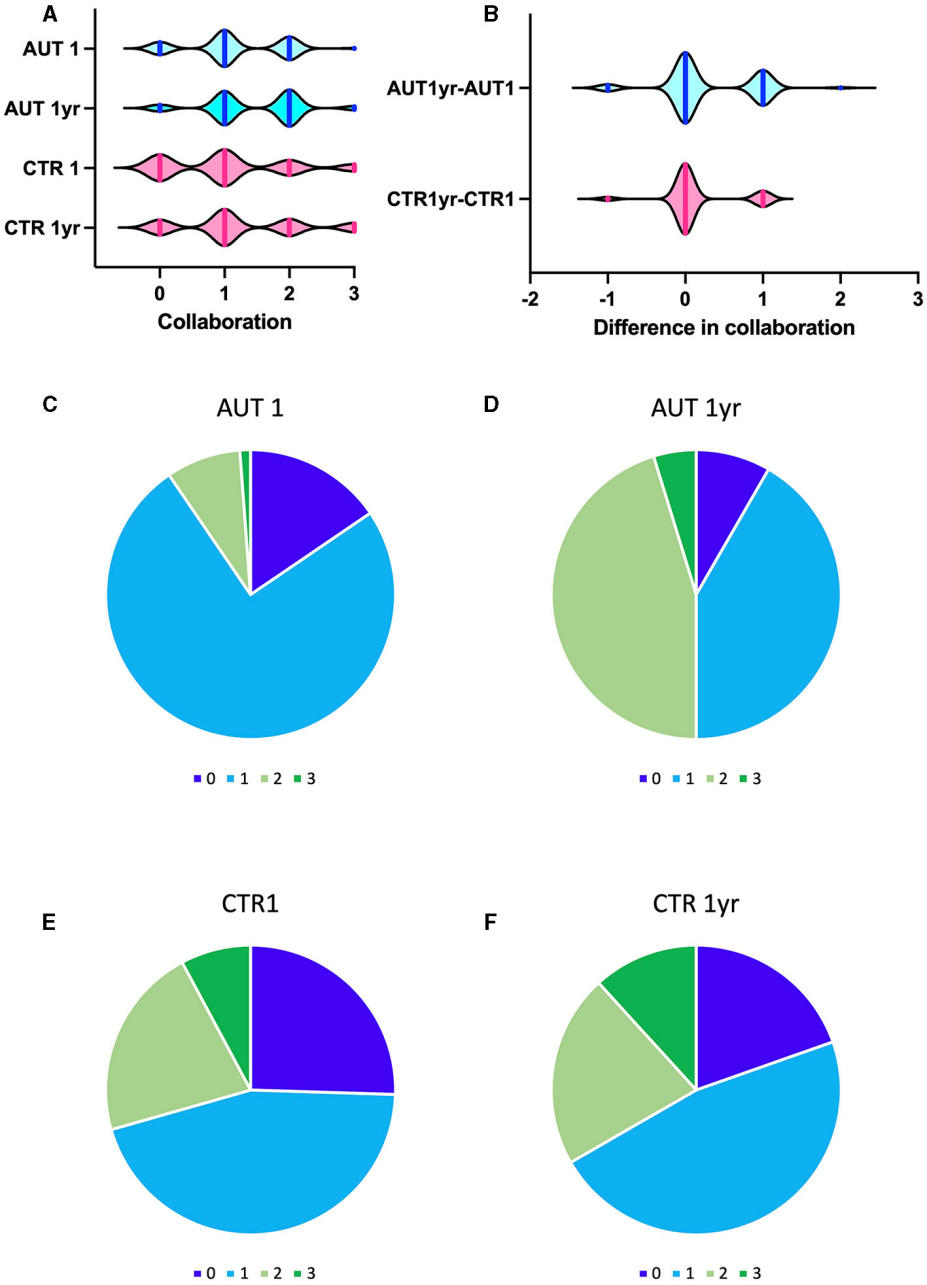
Change in collaboration after 1 year in experimental and control groups. **(A)** Degree of collaboration on a 0–3 scale for AUT1 (experimental group at visit 1); AUT 1 year (experimental group, after 1 year); CTR1 (control group, visit 1); CTR 1 year (control group, after 1 year); the longer the vertical line, the larger the number of patients for that degree of collaboration. **(B)** Difference in collaboration for each patient, 1 year after the onset of the program, compared with the first visit. More patients in the experimental group showed a positive increase in collaboration after 1 year. **(C–F)** Color-coded degree of collaboration: 0 none, blue; 1 scarce, light blue; 2 good, pale green; 3 optimal, green; shades of blue indicate a negative approach, shades of green indicate positive collaboration. **(C, D)** degree of collaboration for the experimental group at the first visit **(C)** and after 1 year, at the sixth visit **(D)** half of the patients show a positive degree of collaboration. **(E, F)** Degree of collaboration for the control group at the first visit **(E)** and after 1 year at the second visit **(F)**.

In order to appreciate the improvement in compliance to the visits, the degree of collaboration was compared with that of a group similar in age, sex, and diagnosis, but that was not enrolled in the 2-month schedule and provided supporting aids. As the major changes in collaboration were already apparent at visit 6 in the experimental group, we compared the degree of collaboration between the two groups at the beginning and after 1 year (visit 6 for the treated group and visit 2 for controls, regardless of other visits that were carried out for emergencies in between). While no difference in collaboration was apparent at the first visit between the groups (U = 3180, *p* = 0.149, Mann–Whitney test), the experimental group experienced a significant improvement in collaboration (W = 368, *p* < 0.0001, Wilcoxon test), while the control group did not (W = 42, *p* = 0.065, Wilcoxon test). Notably, positive collaboration degrees 2 or 3, which allow easy treatment at the dentist chair, changed from 9.52 to 50% in the experimental group but only from 29.41 to 33% in the control group [chi-square 13.942, *p* < 0.0002 (Figure 5)]. Furthermore, the control group included some patients whose collaboration from the beginning was already very good and did not need additional support; hence, they did not enter the program.

In detail, after 1 year, in the experimental group, 61.9% did not change their collaboration degree, 5.95% worsened, and 32.1% improved, while in the control group, 78.4% remained unchanged, 3.9% worsened, and only 17.6% improved their collaboration. In particular, in the experimental group, incisor reconstruction after trauma (in two patients) and professional oral hygiene in all patients were performed without the need for sedation or general anesthesia.

Finally, we asked whether the lockdown experience for COVID-19 (from 8 March 2020 to 28 May 2020 in our country) influenced the collaboration degree. The last appointment before the lockdown was compared with the first after the lockdown (between May and June 2020): Out of 83 patients for which the comparison was possible, no significant change was detected (U = 3,310, *p* = 0.638, Mann–Whitney test). In detail, 71 children maintained the same level, 8 improved by one level, and only 4 worsened by one level.

## 4. Discussion

ASD affects a variety of processes, with underlying common traits: difficulty in social relationships and stereotyped activities, possibly worsened by language difficulties and sensory problems, which often prevent the completion of common daily activities and hinder what can be actually achieved by the affected person. Children with ASD present different behavioral and cognitive signs, for example, in reporting pain that often hides their essential requirements for overall health, which is particularly detrimental in a lifelong perspective. The behavioral difficulties shown by ASD patients may preclude a timely and proper dental treatment, which is often performed under general anesthesia (39), and in some centers, it is never delivered under conscious conditions (40). As some risks, such as those for caries and periodontal disease, are higher in ASD persons (41), oral health maintenance may be guaranteed by preventive strategies, including tooth brushing and regular dentist visits, to discover the disease at its initial appearance. These strategies may also prevent malocclusion or correct the appearance of repetitive behavioral habits (suckling, grinding, and biting) that may negatively affect oral development. Preventive strategies, including sealants and fluoride varnish, are effective in reducing caries risk in autistic patients (42); hence, it is relevant to improve compliance with dental care.

Professionals should be aware of the conditions shown by each patient and of the most powerful techniques for managing them. Dentists may ask for support from other healthcare workers (e.g., speech and language therapists, occupational therapists, and nurses) to instruct caregivers and patients themselves to maintain proper oral hygiene (43). Some basic techniques include collaboration with caregivers, improved communication skills, distraction, imitation, and desensitization of the patient, and use of specific tools and technologies including sedation or general anesthesia. Each of them should be used when needed and adapted to the patient's capability for obtaining collaboration: This is the base for continuing dental cures for a lifetime (44, 45). Behavioral intervention with visual tools, either video or photograph, has been successfully used to prepare children for the first dental examination (46) and for tooth brushing (47) and, either culturally adapted or not, may improve oral health status (48). Furthermore, virtual reality and video modeling have been used successfully to improve oral hygiene (49, 50), and apps have been found to be more effective than pictures in accepting some procedures (51). Even parents' training has been implemented to ameliorate oral hygiene (52). In the present study, we used both parent training and visual pedagogy, in addition to an extended duration of interaction with professionals, to improve collaboration over a prolonged timeframe. The strategy of combining different techniques and tailoring them to the changing behavior of children was the key to success. It may represent a cost-effective plan to improve overall health and promote healthy development, aimed at oral health maintenance throughout life, by increasing acceptance and inclusion of dental visits in the life routine of ASD persons. According to parents' reports, up to one-third of ASD children may undergo dental treatment under general anesthesia (53). One study reports the use of desensitization to accustom ASD children to visit, an ability that is still present after 2 years, while 22% still required general anesthesia (54), a percentage that is in line with what we found here for most invasive treatments, including difficult fillings, extraction or root canal therapy, or oral surgery. Our data show that ASD should not preclude access to dental care since even young children may be accustomed to the dental chair, which may improve their compliance with oral healthcare at home and acceptance, often without the need for sedation also for invasive maneuvers such as professional oral hygiene, sealings, and fillings. The main limitation of this study is the lack of additional strategies for children refractory to the interventions; hence, dental treatment was possible only under general anesthesia in some cases. Moreover, additional analyses could be implemented to account for variability, like advanced regression multilevel for hierarchic random effects, yet we believe that sticking to raw findings can still shed some light on clinical outcomes. Out of the total number of dental treatments during this project, nearly half was done at the dentist's chair, thus reducing costs for general anesthesia and avoiding unnecessary exposure of children to potentially harmful treatments. Only a minority of children, mainly during the first visits, refused to enter the operations room and open their mouths, yet there was some rejection of the dental mirror. Surprisingly, the light, which is a strong sensory stimulus, was much more accepted than the towel: This finding warns us to closely monitor children's behavior because their perception of sensory stimuli may differ from ours. Notably, the degree of collaboration improved during the 1^st^ year and remained the same for the actions preceding the visit, the hygiene, and the dentist examination. In control children, such a large improvement did not take place. The onset of the pandemic did not negatively influence access and compliance to the examination, despite the harsh procedures to access the department (queueing, temperature measurements, and people wearing facial masks). Hence, we underline the strength of the achievements, which appear resilient to the changed regulations for hospital access and use of personal protective equipment. This is relevant considering the lifelong management of ASD patients in the dental setting, which may improve and maintain their overall health.

## Data availability statement

The original contributions presented in the study are included in the article/Supplementary material, further inquiries can be directed to the corresponding authors.

## Ethics statement

The studies involving humans were approved by Ethical Committee of the Province of Padova, CESC Code 4578/U6/18. The studies were conducted in accordance with the local legislation and institutional requirements. Written informed consent for participation in this study was provided by the participants' legal guardians/next of kin.

## Author contributions

IP: Conceptualization, Investigation, Writing—review & editing. EB: Investigation, Writing—review & editing. GM: Investigation, Writing—review & editing. FB: Writing—review & editing, Formal analysis, Validation. CG: Writing—review & editing, Conceptualization, Funding acquisition, Supervision. CM-C: Conceptualization, Supervision, Data curation, Formal analysis, Methodology, Visualization, Writing—original draft.
