# Supplementary material for: Behavioral guidance for improving dental care in autistic spectrum disorders
Source: Front Psychiatry. 2023 Nov 14;14:1272638. doi: 10.3389/fpsyt.2023.1272638 (PMC10682214; doi:10.3389/fpsyt.2023.1272638)
Supplement: Supplementary file 1 [file Data_Sheet_1.PDF]

Supplementary Material

SUPPLEMENTARY MATERIAL
Pastore et al.

Supplementary S1

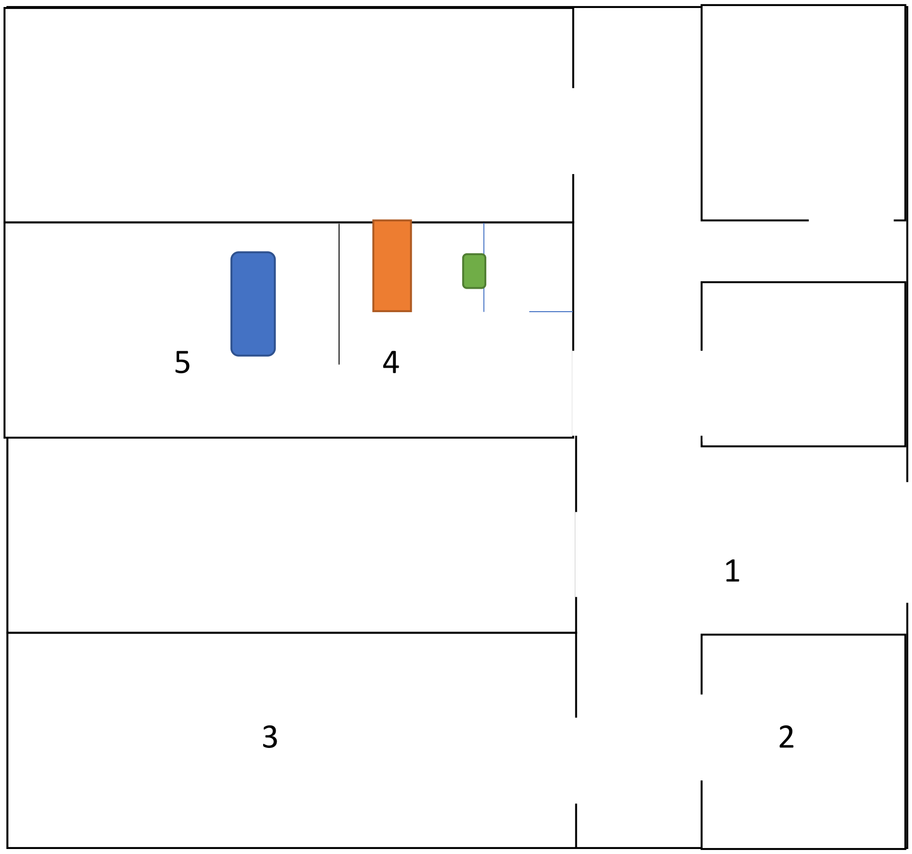

Supplementary Figure S1. A schematic drawing of the Department of Community Dentistry. 1: main entrance; 2: Tea room; 3: waiting room: children were allowed to use this room if needed; 4: office (orange: desk; green: basin): 5: operating room (blue: dentist's chair).

Supplementary S2

Details of the enrolled patients in the experimental and control groups. Red: 2 patients that quit the project after the first appointment (without dentist visit). Yellow: patients that completed less than 15 appointments in the experimental group.

Year of birth	Sex M/F	N° appointments		CONTROLS	Year of birth	Sex M/F	N° appointments
2012	M	10			2012	M	7
2010	M	13			2014	M	5
2010	M	18			2013	M	2
2007	F	20			2012	F	8
2010	M	15			2012	F	3
2008	M	15			2014	M	5
2009	M	15			2013	M	2
2010	M	18			2010	M	4
2012	M	17			2013	M	2
2012	F	18			2007	M	8
2012	M	20			2014	M	6
2013	M	10			2012	M	5
2012	M	19			2009	M	4
2013	M	15			2007	M	2
2015	F	0			2006	M	3
2010	F	18			2011	M	3
2011	M	20			2013	M	3
2010	F	18			2010	F	4
2008	M	20			2013	M	5
2008	M	17			2013	M	7
2011	M	0			2012	M	2
2014	M	20			2012	F	9
2012	M	18			2009	M	1
2008	M	18			2010	F	7
2009	M	19			2011	M	1
2009	M	19			2012	M	2
2013	M	17			2013	M	2
2013	M	17			2007	F	6
2013	M	19			2009	M	3
2006	F	13			2014	M	2
2013	F	19			2015	M	2
2014	M	19			2013	M	2
2014	M	17			2006	F	18
2009	M	18			2011	M	1
2012	M	17			2011	M	1
2008	M	16			2009	M	6
2009	F	16			2012	M	5
2012	F	20			2009	M	1
2007	M	19			2014	M	1
2011	F	20			2008	M	30
2013	F	15			2007	M	6
2007	M	21			2014	M	4
2010	M	18			2009	M	6
2010	M	22			2008	M	23
2011	M	17			2012	M	3
2006	M	15			2013	M	1
2009	M	15			2012	M	2
2012	M	19			2014	M	1
2009	M	6			2008	M	10
2006	M	15			2011	F	1
2010	M	15			2015	M	2
2009	M	11			2013	F	1
2015	M	18			2012	M	5
2009	M	22			2011	M	6
2009	M	20			2008	M	1
2008	M	15			2006	M	3
2011	M	18			2009	F	1
2012	M	22			2007	M	1
2015	M	12			2011	M	2
2013	M	15			2009	M	5
2013	M	17			2013	M	4
2013	M	17			2009	M	2
2014	M	18			2012	M	1
2012	M	21			2011	M	1
2013	F	17			2010	M	4
2009	M	13			2009	F	2
2010	M	11			2007	M	12
2014	M	17			2006	M	5
2013	M	19			2014	F	5
2011	M	17			2006	M	14
2012	M	18			2006	M	10
2009	M	19			2006	M	12
2009	M	14			2015	M	3
2014	M	19			2011	M	3
2013	M	19			2012	F	1
2009	F	16			2014	M	2
2011	M	17			2015	M	1
2010	M	19			2010	M	1
2013	M	17			2013	M	1
2011	M	19			2013	M	1
2007	M	18			2006	M	8
2012	M	13			2006	M	10
2014	M	22			2015	M	3
2012	F	18			2011	M	2
2011	M	18					
2012	M	18					

Supplementary S3

An example of the figures used for visual pedagogy on toothbrushing. A banner was sticked over the basin, as a visual aid. If needed, these were used at the second appointment, the procedure was tried in the washbasin present in the office and later at home, with the aid of the images.

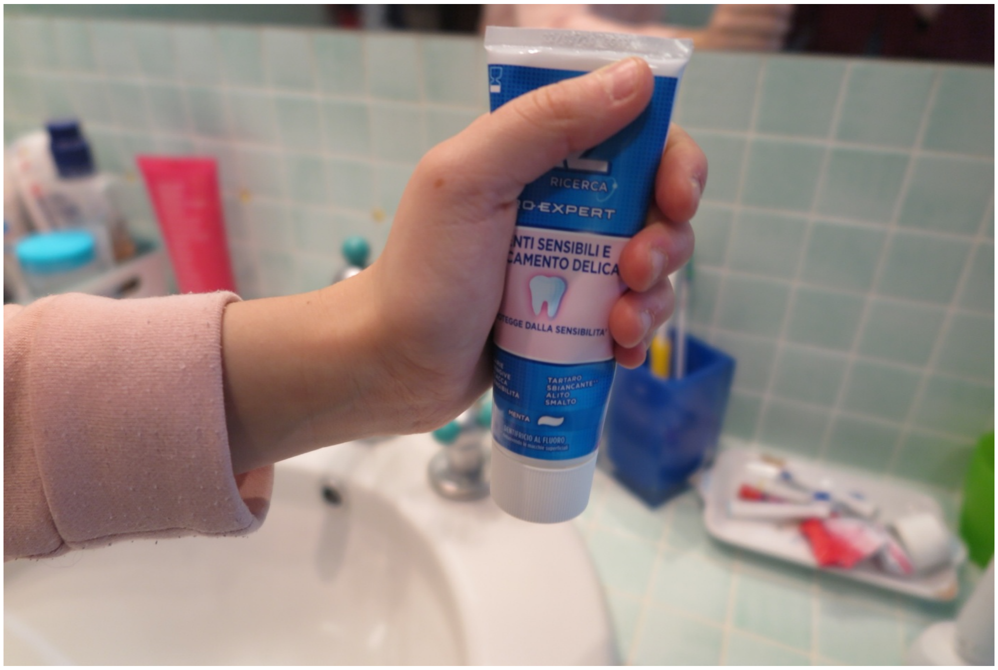

TAKE THE TOOTHPASTE

Supplementary S4

At every appointment, the following information was collected, coded and stored in a secure file:

1. Patient’s data:
    1) Patient’s code, name, gender, birthdate
    2) Type of visit: with the dentist or with the professionals for oral hygiene
    3) Date of the appointment
    4) Overall collaboration: on the Frankl 0-3 scale (Frankl et al. 1962): 0 clearly negative (oppositive, does not accept any direct contact), 1 moderately negative (oppositive for most of the time, hardly accepts some contact), 2 moderately positive (accepts some but not all the maneuvers), 3 clearly positive (accepts all procedures)

2. General anesthesia (if relevant)
    1) General Anesthesia: yes/no
    2) Date
    3) Code for the treated teeth
    4) Number of treated teeth

3. Access to the dentist's room (items 3. and 4. were added after the adoption of regulations for COVID-19 prevention)
    1) Enters into the hospital: No/yes (always accompanied by caregivers)
    2) Enters into the department: No/ yes (voluntarily)/yes (supported by caregivers)
    3) Measurement of temperature (from April 2020, necessary for COVID-19 surveillance): Refused/yes/not requested (before April 2020)
    4) Wears the facial mask (see c.): No/yes/not requested
    5) Seats in the office: No (refused)/yes/ not requested (because the patient can enter the operation room)
    6) Voluntarily enters the operations room: No/Yes/Yes-guided by caregivers
    7) Type of visit: No (no procedure possible)/can be visited while standing/can be visited in caregiver's arms/seats in the dentist's chair with difficulty/seats in the dentist's chair without difficulty/Visit on pushchair
4. Dental treatment:
    1) Towel: No (active rejection)/yes/not necessary (e.g., because no visit was possible)
    2) Opens the mouth: No/yes/ with difficulty
    3) Light: No/yes/not necessary (e.g., because no visit was possible)
    4) Use of his/her own toothbrush: No/yes/not necessary
    5) Visit using only dentist's hands: No/yes/not necessary
    6) Mirror: No/yes
    7) Dentist's probe: No/yes/not necessary
    8) Air-water syringe: No/yes/not necessary
    9) Sum of accepted maneuvers
5. Professional oral hygiene:
    1) Maneuvers: No (active rejection)/his-her own toothbrush/contra-angle handpiece without polish/contra-angle with polish/non-spinning contra-angle/not necessary or not attempted
    2) Gauze and chlorhexidine: No/yes/not necessary or not attempted
    3) Rubber points: No/yes/not necessary or not attempted
    4) Excavator: No/yes/not necessary or not attempted
    5) Dental scaler: No/yes/not necessary or not attempted
    6) Saliva suction: No/yes/not necessary or not attempted
6. Sealings:
    1) Code for treated teeth
    2) Total number of treated teeth
    3) Mirror: No/yes
    4) Probe: No/yes
    5) Tweezers: No/yes
    6) Cotton rolls: No/yes
    7) Saliva suction: No/yes
    8) Etchant: No/yes
    9) Sealant: No/yes
    10) Curing light: No/yes
7. Temporary fillings:
    1) Code for treated teeth
    2) Total number of treated teeth
    3) Mirror: No/yes
    4) Probe: No/yes

5) Tweezers: No/yes
6) Cotton rolls: No/yes
7) Saliva suction: No/yes
8) Excavator: No/yes
9) Turbine: No/yes
10) Contra-angle handpiece: No/yes
11) Temporary cement: No/yes (without problems)/yes (hardly accepted)

8. Permanent fillings (composite):
    1) Code for treated teeth
    2) Total number of treated teeth
    3) Mirror: No/yes
    4) Probe: No/yes
    5) Tweezers: No/yes
    6) Cotton rolls: No/yes
    7) Composite (in syringe): No/yes/not necessary
    8) Composite (in tube): No/yes/not necessary
    9) Turbine: No/yes
    10) Contra-angle handpiece: No/yes
    11) Curing light: No/yes
9. Extractions:
    a. Extractions: No/yes
    b. Code for teeth:
    c. Number of teeth:
    d. Local anesthesia: No/yes

Supplementary S5

A

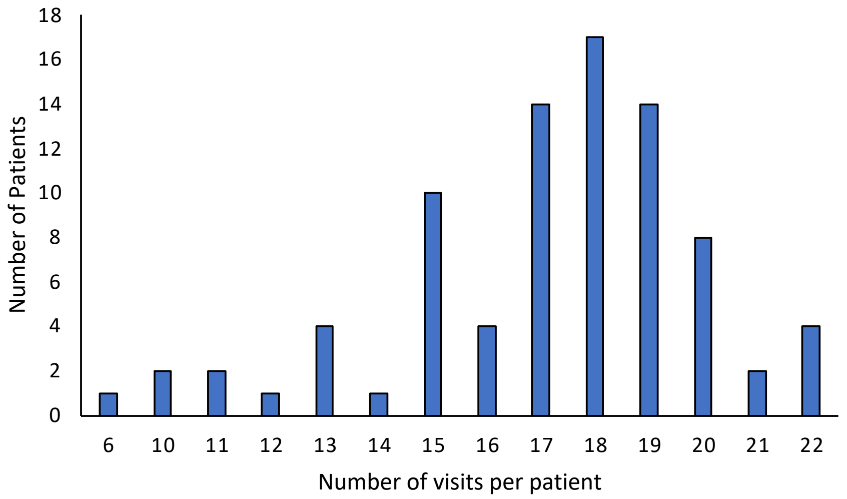

B

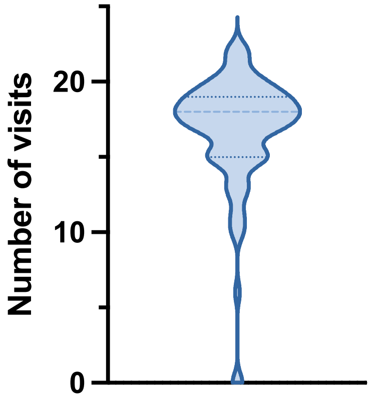

C

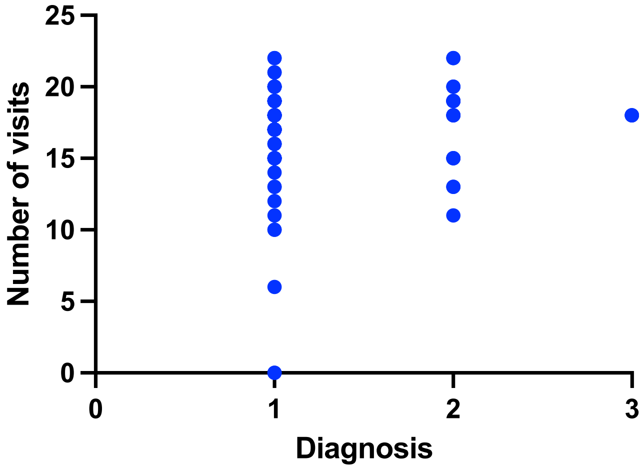

S5A: Number of visits received by patients. This indicates the degree of compliance to the two-month schedule, according to the number of visits done by each patient in the three-year period. The majority of patients adhered to 15 visits or more.

S5B: Number of visits plotted against the number of patients; the larger the violin the greater the number of patients. The dashed line is the mean and the dotted lines 95% CI.

S5C: Number of visits plotted against diagnosis: 1: ASD, 2: PDD-NOS, set according to DSM-4 (from DSM5, PDD-NOS is part of ASD) 3: ASD in addition to other syndromes (e.g.: Rett syndrome).

Supplementary S6

A

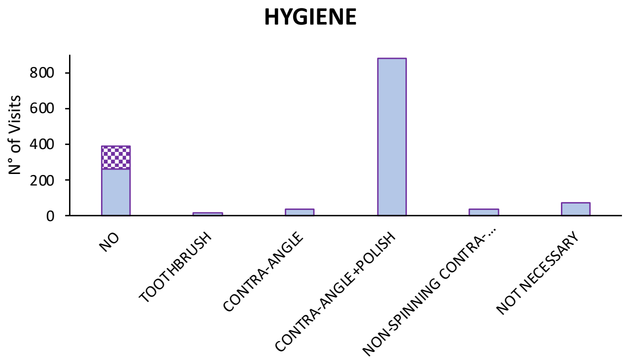

B

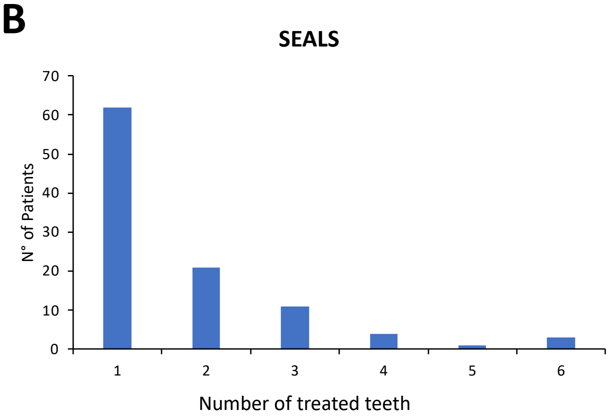

C

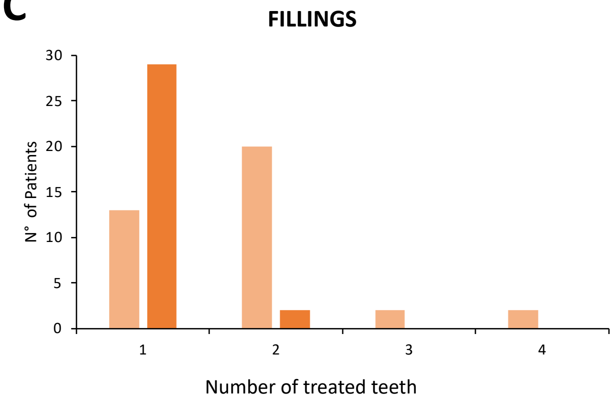

S6 A. Number of times one instrument was accepted for professional oral hygiene. No: instruments were refused, the checked part of the bar indicates that use of instrument was prevented for safety reasons related to COVID-19 prevention. Not necessary: after visual inspection, professional hygiene was not required.

S6 B. Number of patients whose teeth were sealed at the dentist's chair, plotted against the number of sealed teeth per patient.

S6C. Number of patients whose teeth were filled at the dentist's chair, plotted against the number of filled teeth per patient. Pale orange: temporary fillings, dark orange: permanent fillings.

Supplementary S7

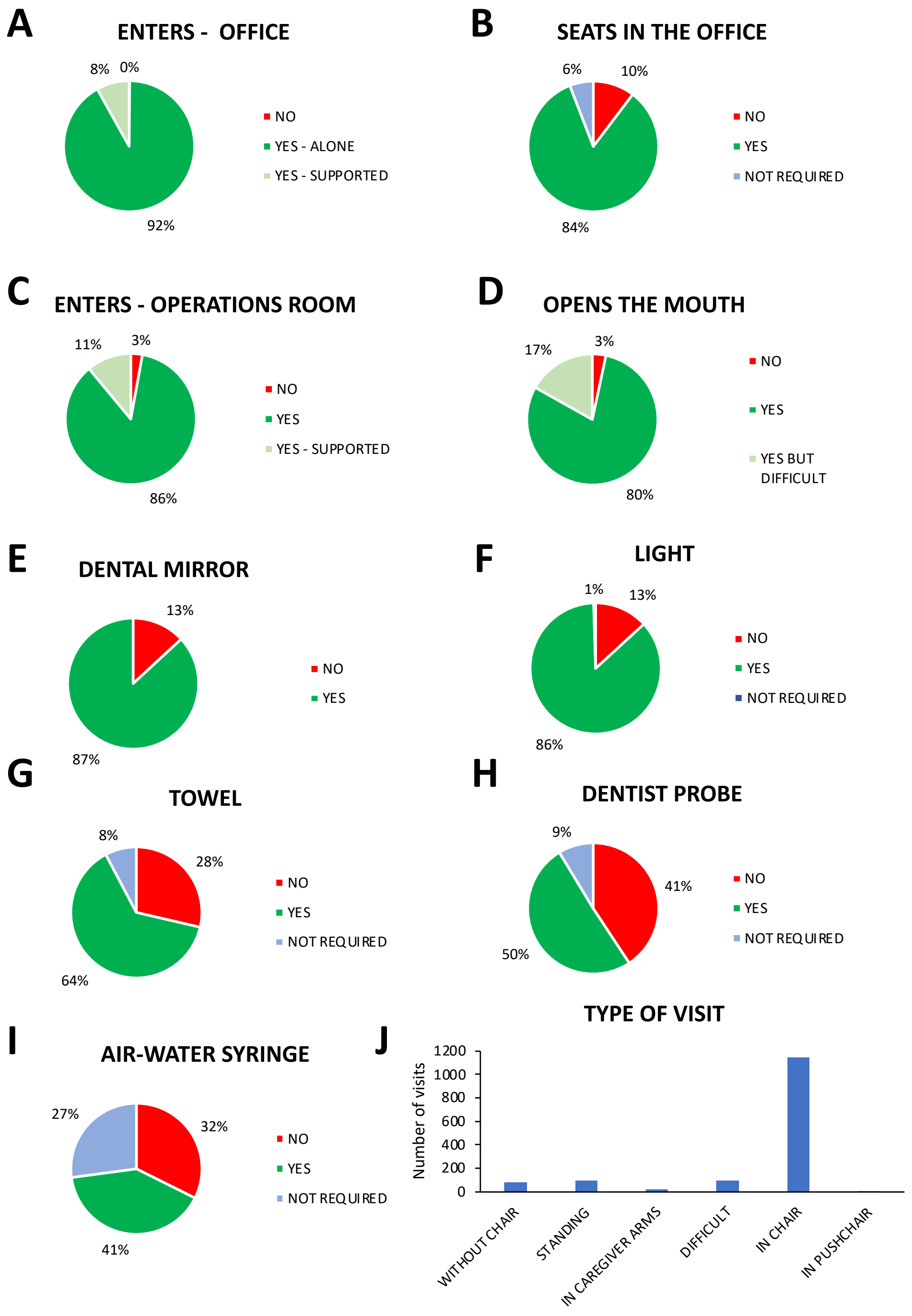

S7. Accepted tools and actions during the appointments. The percentage of visits for each option is shown near the relevant sector. For graphs **A**. to **I**.: red: No (action/instrument refused), green: Yes (action/instrument accepted), light blue: action or instrument not required. **A.** The patient enters the office alone (green), supported by caregivers (pale green), or does not enter (less than 1%, red, not visible). **B**. after entering the office, the patient sits down there. **C.** after entering the office, the patient goes to the operations room. Pale green: the patient needs the support of caregivers. **D.** The patient opens the mouth. Pale green: yes, but with difficulty. **E.** Use of dental mirror. **F.** Use of light. **G.** Acceptance of the towel on the neck. **H.** Acceptance of dentist's probe. **I**. use or air-water syringe. **J.** modality in which the visit was carried out: without chair, standing, in caregivers' arms, done with difficulty (mixed modes), in the dentist's chair, or in the pushchair.
